# Ascorbic Acid Promotes Functional Restoration after Spinal Cord Injury Partly by Epigenetic Modulation

**DOI:** 10.3390/cells9051310

**Published:** 2020-05-25

**Authors:** Jin Young Hong, Ganchimeg Davaa, Hyunjin Yoo, Kwonho Hong, Jung Keun Hyun

**Affiliations:** 1 Department of Nanobiomedical Science & BK21 PLUS NBM Global Research Center for Regenerative Medicine, Dankook University, Cheonan 31116, Korea; vrt23@naver.com (J.Y.H.); ganchimeg12@gmail.com (G.D.); 2 Institute of Tissue Regeneration Engineering (ITREN), Dankook University, Cheonan 31116, Korea; 3 Department of Stem Cell & Regenerative Biotechnology, Konkuk University, Seoul 05029, Korea; hyunjinyoo7@gmail.com; 4 Department of Rehabilitation Medicine, College of Medicine, Dankook University, Cheonan 31116, Korea; 5 UCL Eastman-Korea Dental Medicine Innovation Centre, Dankook University, Cheonan 31116, Korea; 6 Wiregene, Co., Ltd., Cheonan 31116, Korea

**Keywords:** spinal cord injury, demethylation, ascorbic acid, neuronal plasticity, 5-hydroxymethylcytosine

## Abstract

Axonal regeneration after spinal cord injury (SCI) is difficult to achieve, and no fundamental treatment can be applied in clinical settings. DNA methylation has been suggested to play a role in regeneration capacity and neuronal growth after SCI by controlling the expression of regeneration-associated genes (RAGs). The aim of this study was to examine changes in neuronal DNA methylation status after SCI and to determine whether modulation of DNA methylation with ascorbic acid can enhance neuronal regeneration or functional restoration after SCI. Changes in epigenetic marks (5-hydroxymethylcytosine (5hmC) and 5-methylcytosine (5mC)); the expression of Ten-eleven translocation (*Tet*) family genes; and the expression of genes related to inflammation, regeneration, and degeneration in the brain motor cortex were determined following SCI. The 5hmC level within the brain was increased after SCI, especially in the acute and subacute stages, and the mRNA levels of *Tet* gene family members (Tet1, Tet2, and Tet3) were also increased. Administration of ascorbic acid (100 mg/kg) to SCI rats enhanced 5hmC levels; increased the expression of the Tet1, Tet2, and Tet3 genes within the brain motor cortex; promoted axonal sprouting within the lesion cavity of the spinal cord; and enhanced recovery of locomotor function until 12 weeks. In conclusion, we found that epigenetic status in the brain motor cortex is changed after SCI and that epigenetic modulation using ascorbic acid may contribute to functional recovery after SCI.

## 1. Introduction

Axonal regeneration following spinal cord injury (SCI) is difficult to achieve, and no fundamental treatment can be applied in clinical settings. Epigenetic regulation mechanisms are important for the development of the central nervous system (CNS), and interactions with environmental factors can change gene expression [1]. Epigenetic changes may play roles in the regeneration capacity of damaged neurons in the adult brain, as well as in neuronal growth in the developing brain; however, the supraspinal mechanism of epigenetic changes after SCI is still elusive. Previous studies have revealed that histone acetylation and changes in the folate pathway affect learning, memory, neuroplasticity, neurodegeneration, and neuronal regeneration. Mammalian DNA methylation at the 5-position of the cytosine base (5mC) plays critical roles in modulating gene expression, and in regulating cellular and developmental processes [2]. The 5mC can be hydroxylated into 5-hydroxymethylcytosine (5hmC) by the Tet enzyme family [3]. TET dioxygenases depend on ferrous iron and 2-oxoglutarate (2OG), and are members of a large family of enzymes that catalyze a wide range of oxidative reactions; specifically, these enzymes are catalysts for epigenetic modifications of DNA and histones [2].

Ascorbic acid (AA; or its anionic form, ascorbate) is well known as an antioxidant, free radical scavenger, and catalyst in various enzymatic reactions, as it acts as an electron donor to regulate the redox states of iron-containing enzymes [4]. DNA methylation and histone methylation are the main epigenetic modifications in the mammalian genome, and some of the nuclear dioxygenases need ascorbate as a cofactor to catalyze these modifications [5]. For example, previous experiments have shown that ascorbate acts as a cofactor for TET dioxygenases in the conversion of 5mC to 5hmC, as well as to 5fC and to 5caC, which results in DNA demethylation. It has been proposed that the beneficial effects of ascorbate on cancer might be mediated by the induction of 5hmC generation in cancer cells [5]. AA levels are regularly maintained in the central nervous system (CNS) under normal conditions. However, large losses of AA from SCI contribute to disruption of spinal cord function [6]. Previous studies have shown that high-dose AA administration significantly improves the functional recovery of SCI rats [7], and ameliorates blood–brain barrier disruption and mitochondrial alterations in a mouse Alzheimer’s disease model [8]. In addition, other evidence has shown that AA provides protective effects against SCI-induced kidney damage through its antioxidant and anti-inflammatory functions [9]. In this study, we aimed to delineate the serial epigenetic changes that occur within the brain at various time points after SCI, from the acute to chronic stages. We hypothesized that AA may enhance the TET-mediated oxidation of 5mC. Besides a favorable effect of AA on the SCI recovery by reducing the reactive oxygen species (ROS) level, our findings may highlight another mechanism by which AA administration enhances SCI recovery by modulating DNA methylation in the brain after SCI in rats.

## 2. Materials and Methods

### 2.1. Animal Models

All procedures were approved by Dankook University’s Institutional Animal Care and Use Committee (Approval No. 16-045). The animals were housed individually in standard rat cages under a 12-h light/dark cycle with free access to food and water in a temperature-controlled environment (23–25 °C) under controlled humidity (45–50%). Adult female Sprague-Dawley rats (12 weeks old, weighing 230–250 g) were used in this study. The surgical procedure for SCI was performed based on a previously described method [10]. Briefly, the animals were anesthetized with 2% isoflurane (Choongwae Pharma, Seoul, Korea) in 2:1 N_2_O:O_2_ in an anesthesia chamber. T9 laminectomy was performed to expose the dorsal surface of the spinal cord. A moderate contusion injury (200 kdyn) was performed using an Infinite Horizon impactor (Precision Systems Instrumentation). Sham-operated control rats underwent laminectomy alone at the T9 level without contusion injury. After SCI, the muscle and skin were closed in layers with 5-0 silk sutures. Cefotiam hydrochloride (40 mg/kg, Hanmi Pharma, Seoul, Korea) and acetaminophen syrup (10 mg/kg, Janssen Pharmaceutica, Titusville, NJ, USA) were administered to all operated rats for three days to prevent bacterial infection and control postoperative pain. Bladder expression was performed twice a day manually until spontaneous bladder expression was recovered.

### 2.2. Preparation and Administration of AA

AA (Jeil Pharm, Seoul, Korea) was administered intraperitoneally at a dose of 5, 100, or 200 mg/kg daily, beginning 30 min after SCI and continuing until 3 months after SCI, just before sacrifice. The control group was given the same volume of distilled water intraperitoneally.

### 2.3. Histology

Sham-operated or SCI rats from each group were deeply anesthetized and transcardially perfused with 200 mL 0.9% normal saline followed by 4% paraformaldehyde (Hushi Inc., Shanghai, China) in 0.1 M PBS (pH 7.4) via a peristaltic pump. The brains and thoracic spinal cords were removed, postfixed overnight in 4% paraformaldehyde at 4 °C, and cryopreserved in 30% sucrose solution at 4 °C for 3 days. The brains and spinal cords were embedded in M1 compound (Thermo Fisher Scientific Inc., Waltham, MA, USA). The spinal cords were then sectioned sagittally at a thickness of 16 µm, and the brains were sectioned in the coronal plane (10 µm thickness) using a cryostat. Hematoxylin and eosin staining (H&E) was performed on slides containing the lesion epicenter to examine the cavity area of the injured spinal cord at different time points. The sections were rinsed with PBS, immersed in hematoxylin solution for 2 min, and then immersed in eosin solution for 10 s. After washing with tap water for 2 min, the sections were dehydrated through a graded ethanol series, cleared with xylene and then imaged with a Nikon inverted microscope (Nikon Corporation, Tokyo, Japan). The lesion cavity area in H&E stained sections (*n* = 4) for each experimental group was measured by manual outline under a light microscope at 100× magnification and calculated using ImageJ software (1.51t, National Institutes of Health, Bethesda, MD, USA) as described in a previous study [11].

For NF200 staining, the sections were washed with PBS and the peroxidase activity was blocked in 0.3% H_2_O_2_ in distilled water for 30 min at room temperature. After rinsing, the sections were incubated in 0.2% Triton-x100 in PBS for 5 min, 2% NGS in PBS for 1 h at room temperature, and then in the primary rabbit anti-NF200 (1:100 Millipore, Darmstadt, Germany) antibody in 2% NGS at 4 °C overnight. Then, the sections were incubated in biotinylated goat antirabbit secondary antibody (1:200 Jackson ImmunoResearch Laboratories, Inc., West Grove, PA, USA) in 2% NGS for 2 h at room temperature, followed by incubation in the Vectastain Elite ABC elite kit (Vector Laboratories, Inc., Burlingame, CA, USA) for 30 min. Lastly, the NF200 staining was revealed with DAB (3,3′-Diaminobenzidine) solution (0.05% 3.3’-diaminobenzidine tetrahydrochloride (Sigma, St. Louis, MO, USA), 0.06% NiCl2 (Sigma), 0.003% H_2_O_2_), then the reaction was stopped by distilled water. The sections were dehydrated, coverslipped, and allowed to dry. The NF200 labeled axon images were captured under 20× and 40× magnification using a microscope (EVOS M7000, Thermo Fisher), the axon intensities were quantified using the image J software as previously described [12], and the intensity was expressed as a relative value to the control group.

### 2.4. Immunohistochemistry (IHC)

IHC was used to analyze epigenetic changes within the brain and the inflammatory responses of the contused spinal cord. Frozen sections were incubated with 0.2% Triton X-100 in 1X PBS solution for 5 min, washed with 1× PBS, and blocked with 2% normal goat serum in 1× PBS for 1 h. Primary antibodies were diluted in 2% normal goat serum and the slides were incubated with the antibodies overnight at 4 °C. The primary antibodies used were as follows: rabbit anti-5hmC (1:500, Active Motif, Carlsbad, CA, USA), mouse anti-5mC (1:500, Active Motif), mouse anti-NeuN (1:100, Millipore), rabbit anti-NeuN (1:1000, Abcam, Cambridge, MA, USA), guinea pig anti-NeuN (1:500, Synaptic Systems, Goettingen, Germany), mouse anti-glial fibrillary acidic protein (GFAP) (1:1000, Sigma), rabbit anti-GFAP (1:1000, Dako Cytomation, Carpinteria, CA, USA), mouse antimonocyte or -macrophage ED1 (1:400, Millipore), rabbit anti-5HT (1:2000, Sigma), rabbit anti-Tet1 (1:100, Abcam), rabbit anti-Tet2 (1:100, Millipore), and rabbit anti-Tet3 (1:100, Santa Cruz Inc., Dallas, TX, USA). The slides were washed in PBS and incubated for 2 h with fluorescent secondary antibodies (FITC-, Alexa 594-, Alexa 647-conjugated, Jackson ImmunoResearch) at 1:200 dilutions in 2% normal goat serum. The sections were washed three times for 5 min with PBS, mounted with fluorescence mounting medium (Dako), and imaged using confocal microscopy (Carl Zeiss Inc., Oberkochen, Germany).

Mature neurons in layers IV and V of the primary motor cortex (M1) were analyzed for DNA methylation and demethylation. For quantification of 5hmC and 5mC fluorescence intensity, three representative images in the primary motor cortex (M1) per animal were captured at 200× magnification; all images were obtained with fixed acquisition settings via confocal microscopy. The average intensity of 5hmC or 5mC costaining with NeuN was measured using ImageJ software (v. 1.51t, National Institutes of Health). The intensity of TET family protein staining was analyzed in a similar fashion. For quantitation of inflammatory responses in vivo, images of ED1-positive cells at the lesion site in three sagittal sections were selected and visualized using a confocal microscope at 100× magnification. ED1-positive macrophages were counted manually within the lesion cavity and expressed as cell number per 1 mm^2^ [10].

### 2.5. Axon Quantification

Axons labeled for IHC were quantified using NIH ImageJ analysis software (version ImageJ 1.51t, National Institutes of Health), and all images were captured using a confocal microscope (Zeiss) at 20× magnification. Three sagittal sections through the lesion epicenter containing the most axon-dense area for 5HT were selected per rat and GFAP antibody double labeling was used to outline the lesion border [13]. To quantify 5HT axons, five images were randomly selected from fields in the rostral, central, and caudal areas of the lesion site. A constant threshold value was used for every image and values from five different 5HT axons were averaged for each field to measure the percentage of the intensity and density of axons using the Image J software (1.51t, National Institutes of Health) as previously described [14,15].

### 2.6. Functional Assessment

The locomotor recovery of paralyzed hindlimbs after SCI was assessed with the Basso, Beattie, and Bresnahan (BBB) locomotor rating scale and horizontal ladder walk test (*n* = 10 per group). The BBB scale ranges from 0 points (no hindlimb movement) to 21 points (normal hindlimb movement) [16]. The rats were evaluated by two examiners, who were blinded to the identities of the rats. The hindlimb movements of the rats were observed for 4 min in an open field (cylindrical-shaped acrylic box, 90 cm in diameter and 15 cm high, with a smooth floor). The horizontal ladder test was performed on a runway made of acrylic walls (10 cm tall, 127 cm long, 8 cm between walls, 1 cm between rungs) as previously described [17]. All rats were trained several times before surgery to achieve crossing of the beam with no more than one forepaw misplacement for five consecutive trials. After SCI, each rat was given only one trial from 1 week after injury and the walk was video-recorded with a digital camcorder. The ladder score was calculated as follows: ladder score = erroneous steps of hindlimb/total steps of hindlimb × 100 (%) [17]. The locomotor function was examined every 7 days until sacrifice.

### 2.7. Genomic DNA Preparation and Dot Blot Assay

Genomic DNA was extracted from the cerebral cortexes of the rats in each group and isolated with a DNeasy Blood (Qiagen, Hilden, Germany) and Tissue Kit (Qiagen) for quantification of 5hmC and 5mC within genomic DNA (*n* = 3 per group). Purified genomic DNA samples were spotted on a nitrocellulose membrane (0.2 µm pore size) using a 96-well manifold apparatus (Manifold I; Schleicher and Schuell, Dassel, Germany). The DNA was immobilized to the membrane by baking at 80 °C for 2 h. The membrane was then blocked with 5% skim milk and incubated with mouse anti-dsDNA (1:2000, Abcam), rabbit anti-5hmC (1:2000, Active Motif), or rabbit anti-5mC (1:1000, Active Motif) at room temperature (RT) for 1 h, incubated with an antimouse or antirabbit horseradish-peroxidase-conjugated antibody (Jackson) for 1 h at RT, and antibody binding was visualized by enhanced chemiluminescence (ECL). The relative amounts of 5hmC and 5mC in genomic DNA were calculated using ImageQuant TL software (GE Healthcare, v 7.0, Chicago, IL, USA) as previously described [18].

### 2.8. RNA Isolation, Quantitative Real-Time PCR (qRT-PCR), and Functional Enrichment Analysis

Global changes in the mRNA expression of genes related to inflammation (cell adhesion molecule 44 (Cd44), interleukin (IL)-1 beta (IL-1β), IL-6, tumor necrosis alpha (TNFα)), regeneration (Krüppel-like factor–4 (KLF4), suppressor of cytokine signaling 3 (Socs3), insulin-like growth factor-1 (IGF-1), midkine (Mdk), mitogen-activated protein kinase 14 (MAPK14), nuclear factor erythroid 2-related factor 2 (Nrf2), tubulin alpha-1A (Tuba1a), and Wnt-4 (Wnt4)), degeneration (Rho-associated protein kinase 2 (Rock2), semaphorin 3A (Sema3a), phosphatase and tensin homolog deleted on chromosome 10 (PTEN), and Wnt-5a (Wnt5a)), and of Tet family genes (Tet1, Tet2, and Tet3) in the motor cortex were evaluated using qRT-PCR (*n* = 4 per group). Total RNA was extracted from the cerebral cortexes of the rats in each group using RNeasy Mini Kit (Qiagen, Hilden, Germany) and cDNA was synthesized from 2 mg of total RNA with random hexamer primers and SuperScript ^Ш^ (Invitrogen, Carlsbad, CA, USA). Primers were designed with the UCSC Genome Bioinformatics platform and the NCBI database, and are listed in Table 1. The qRT-PCR was performed with Fast SYBR Green Master Mix (Applied Biosystems, Waltham, MA, USA) on a StepOne Real-Time PCR system (Applied Biosystems). All experiments were carried out in triplicate, and the expression of each gene was normalized to the mRNA expression of the endogenous gene *GAPDH* and calculated as the relative fold change compared to the expression in the control group. In the experiments conducted to determine the optimal concentration of AA for use in SCI models, the mRNA levels of DNA methyltransferase (DNMT) 1, DNMT3a, and DNMT3b were also evaluated using qRT-PCR.

We also performed functional enrichment analysis of the upregulated and downregulated genes for each time series (5 time points for the sham and SCI models: 1 day, 1 h, 1 week, 1 month, and 3 months; 2 time points for the control and AA-100 groups: 1 week and 3 months) using the Metascape online tool (http://metascape.org). The enrichment of Gene Ontology (GO) terms in the biological process, cellular component, and molecular function categories, and of Kyoto Encyclopedia of Genes and Genomes (KEGG) pathways was analyzed with Metascape. We considered GO terms with *p*-values < 0.01, minimum counts of 3, and enrichment factors of >1.5 to be significantly enriched. The most statistically significant term within a cluster was chosen as the one representing the cluster. A subset of enriched terms was selected and used to render a network plot at each time point to determine the relationships among terms, where terms with similarities of >0.3 were connected by edges.

### 2.9. Statistics

All numeric data are reported as the means ± standard errors and IBM SPSS Statistics 25 (International Business Machines Corp., Armonk, NY, USA) was used for analysis. Shapiro-Wilk tests were used to confirm the normal distributions of all the quantitative data. We evaluated significant differences in 5hmC and 5mC intensities from IHC, and in all quantitative data from the dot blot assays and qRT-PCR analyses between the sham controls and the spinal cord contusion models using Mann-Whitney U tests. One-way analysis of variance (ANOVA) and Games-Howell post hoc tests were performed to detect any differences in numerical data from IHC and functional assessments among the control group and the experimental groups receiving three different concentrations of AA. Repeated measures one-way ANOVA was used to compare locomotor function data, including the BBB and ladder test scores, among the control and three experimental groups. Mann-Whitney U tests were also performed to detect any differences in numerical data from the dot blot assays and qRT-PCR analyses between the control group and the experimental group that received the optimal concentration of AA. The *p* values less than 0.05 were considered to indicate statistical significance.

## 3. Results

### 3.1. SCI Induces Alteration of 5mC and 5hmC Levels in the Brain Motor Cortex

**Figure 1:**
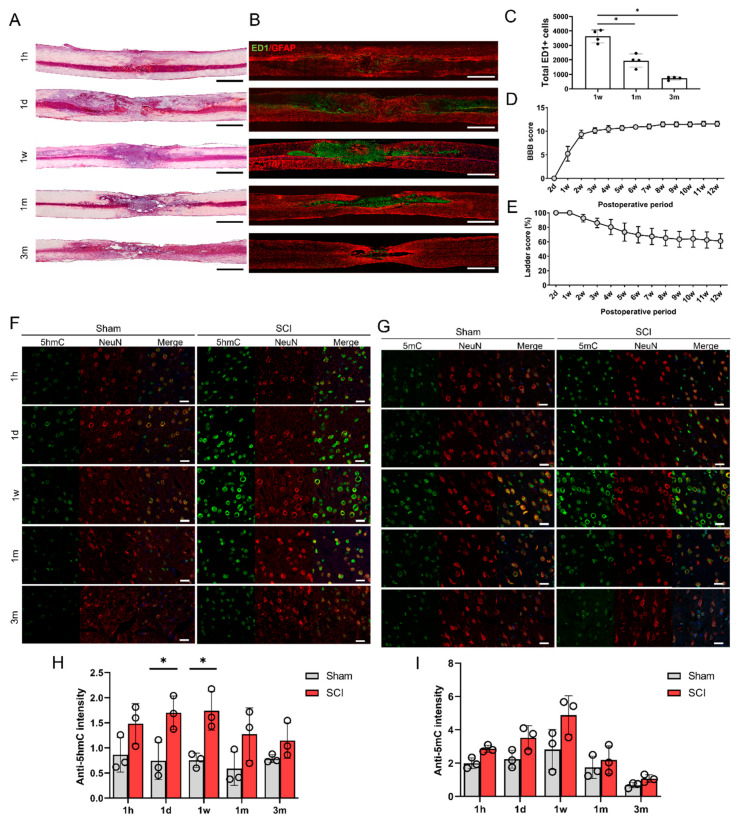
Histological and functional changes following spinal cord contusion injury in rats. (**A**) Representative images of hematoxylin and eosin staining of contused spinal cords at 5 time points (1 h (1 h), 1 day (1 d), 1 week (1 w), 1 month (1 m), and 3 months (3 m)). Black and white scale bars = 1000 µm. (**B**) Representative immunohistochemical images of ED1 (green) and GFAP (red) in contused spinal cords at 5 time points. (**C**) The total numbers of ED1-positive cells within sagittal sections from immunohistochemical images of contused spinal cords at 1 week (1 w), 1 month (1 m), and 3 months (3 m) (*n* = 4 at each time point); * *p* < 0.05 between groups by one-way analysis of variance (ANOVA) and Games–Howell post hoc tests. (**D**,**E**) Basso-Beattie-Bresnahan (BBB) scores (**D**) and ladder scores (**E**) of spinal cord injury (SCI) models from 2 days (2 d) to 12 weeks (12 w) after injury (*n* = 7). (**F**,**G**) Representative immunohistochemical images of double staining of 5hmC (green) and NeuN (red) (**F**), and of 5mC (green) and NeuN (red) (**G**) in the primary motor cortexes of sham control rats and SCI model rats at 5 time points (**G**). Staining of 5mC (**H**) within the primary motor cortexes of sham and SCI rats at 5 time points (*n* = 3 at each time point). White scale bars = 20 µm. (**H**,**I**) The 5hmC (**H**) and 5mC (**I**) intensity in neurons in the primary motor cortexes of sham control (*n* = 3) and SCI model rats (*n* = 3) at 5 time points; * *p* < 0.05 between the sham control and SCI group by the Mann-Whitney U test.

To evaluate the general histologic features of the injured spinal cords from the acute to chronic stages, we performed H&E staining and IHC of the contused spinal cords at five time points: 1 h, 1 day, 1 week, 1 month, and 3 months after SCI. The H&E-stained sections showed a variety of cavitation patterns from 1 h to 3 months after SCI (Figure 1A). Macrophage infiltration into the lesion site was detected by immunohistological staining (Figure 1B), and we found that ED1 + cells were most visible in the lesion cavity 1 week after SCI, and gradually decreased after 1 and 3 months (Figure 1C). We confirmed the standardized behaviors of the moderate spinal cord contusion model animals using BBB and ladder tests. The final BBB score of our moderate SCI model group was 11.56 ± 0.53 twelve weeks after injury (Figure 1D), and the ladder score at 12 weeks was 60.99 ± 9.94% (Figure 1E).

The changes in 5hmC and 5mC levels in the brain were determined in the sham control and SCI groups. To detect epigenetic changes in neurons within the primary motor cortex, neurons double-stained with 5hmC and NeuN or 5mC and NeuN were detected immunohistochemically at 5 different time points in the control and SCI groups (Figure 1F,G). The intensity of 5hmC was significantly higher in the SCI group than in the control group at 1 day (acute stage) and 1 week after injury (Figure 1H). The 5hmC and 5mC intensities in the motor cortex peaked at 1 week after SCI (Figure 1H,I). However, we could not find any differences in 5mC intensity between the sham control and SCI groups (Figure 1I).

### 3.2. Altered 5hmC Levels and Gene Expression in the Brain Motor Cortex after SCI

**Figure 2:**
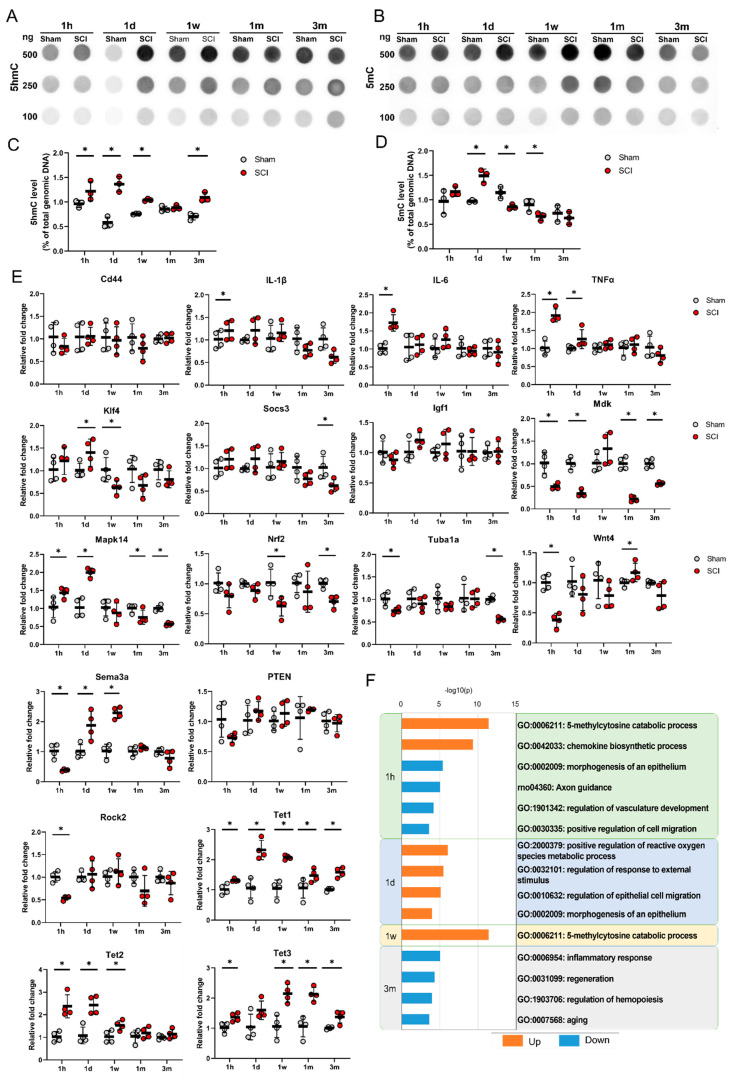
(**A**,**B**) Dot blot and real-time PCR results for the primary motor cortexes of sham control and spinal cord injury (SCI) models at 5 time points. Representative results for 100, 250, and 500 ng of DNA for 5hmC (**A**) and 5mC (**B**) in sham control and SCI model rats at 1 h (1 h), 1 day (1 d), 1 week (1 w), 1 month (1 m), and 3 months (3 m). (**C**,**D**) Quantitative data from dot blot analysis of 250 ng of DNA for 5hmC (**C**) and 5mC (**D**) compared with the amount in total genomic DNA (%) in the sham and SCI groups (*n* = 3 per group); * *p* < 0.05 between the sham control and SCI groups by Mann–Whitney U test at the same time point. (**E**) Quantitative data from real-time PCR for inflammation-related genes (Cd44, IL-1β, IL-6, and TNFα), regeneration-related genes (Klf4, Socs3, Igf1, Mdk, Mapk14, Nrf2, Tuba1a, and Wnt4), degeneration-related genes (Sema3a, PTEN, and Rock2), and TET family genes (Tet1, Tet2, at Tet3) at 5 time points (*n* = 4 per group); * *p* < 0.05 between the sham control and SCI groups by Mann-Whitney U test at the same time point. (**F**) Gene Ontology (GO) functional enrichment analysis was performed for the differentially expressed genes between the sham and SCI groups at 5 time points using Metascape. The values are expressed as the negative log10-transformed *p*-values. Blue bars = upregulated genes, orange color bars = downregulated genes.

The levels of 5hmC and 5mC in the genomic DNA of the motor cortex were determined using a dot blot assay (Figure 2A,B). The 5hmC and 5mC levels for each experiment were normalized to the total amount of dsDNA. We found that the 5hmC levels in 250 ng of genomic DNA were significantly increased at 1 h, 1 day, 1 week, and even 3 months after SCI (Figure 2C). However, the 5mC levels in 250 ng of genomic DNA were increased only at 1 day after SCI and were decreased at 1 week and 1 month after SCI (Figure 2D). We also quantified 500 ng and 100 ng of DNA for 5hmc and 5mc, and similar patterns to 250 ng of DNA were shown (Supplementary Materials, Figure S1), however the optimal intensity, which showed the difference between the control and experimental groups more clearly, was 250 ng of DNA.

The changes in gene expression after SCI were detected using qRT-PCR analysis (Figure 2E). The expression levels of inflammation-related genes, such as IL-1β, IL-6, and TNFα, were significantly elevated in the SCI group compared to the sham group. These genes were transiently upregulated at 1 h and returned to normal expression ranges after the acute stage. In contrast, CD44 gene expression did not differ between the sham and SCI groups. Some regeneration-related genes, including Klf4 and Mapk14, were upregulated in the acute stage, while Socs3, Mdk, Mapk14, Nrf2, and Tuba1a were downregulated in the chronic stage. Some degeneration-related genes, such as Sema3a and Rock2, were downregulated at 1 h, whereas Sema3a was upregulated at 1 day and 1 week after SCI. Igf1 and PTEN were not changed during the entire time period following SCI. Wnt4 was downregulated at 1 h but upregulated at 1 month after SCI. The mRNA levels of Tet1, Tet2, and Tet3 in the brain motor cortex were significantly higher in the SCI group than in the sham group in the acute stage. In particular, Tet1 and Tet3 were upregulated significantly in the SCI group until the subacute and chronic stages, respectively.

Functional enrichment analysis revealed that the upregulated Tet family genes at 1 h and 1 week after injury in the SCI group were related to GO: 006211, which is associated with chemical reactions and pathways resulting in the breakdown of 5mC (Figure 2F). The upregulated genes were also enriched for a GO term related to chemical reactions and pathways resulting in the formation of chemokines (GO: 0042033); however, the KEGG pathway analysis showed that genes downregulated at 1 h were enriched for axon guidance (rno04360) (Figure 2F). Three months after SCI, the genes that were downregulated in the SCI group were enriched for the inflammatory response (GO: 0006954) and regeneration (GO: 0031099) terms (Figure 2F).

### 3.3. AA Improves Functional Recovery and Axonal Sprouting after SCI

**Figure 3:**
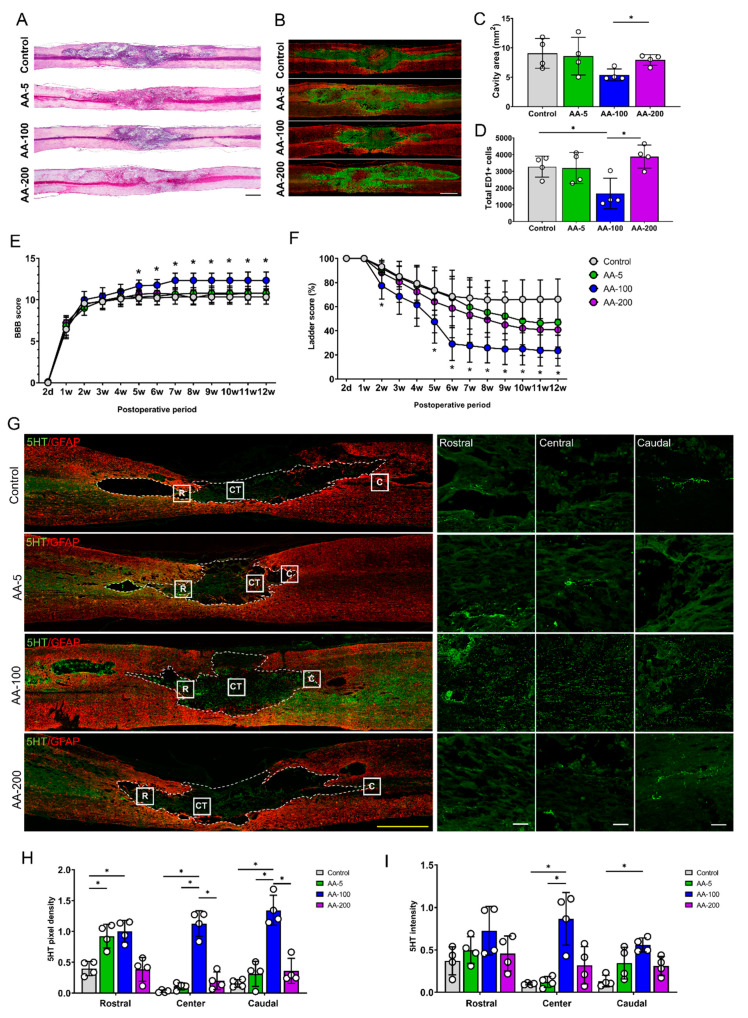
Histological and functional changes after application of ascorbic acid (AA) at concentrations of 5, 100, and 200 mg/kg to SCI models. (**A**,**B**) Representative images of hematoxylin and eosin (H&E) staining (**A**) and immunohistochemical images of ED1 (green) and GFAP (red) (**B**) in contused spinal cords in the control group and the 5, 100, and 200 mg/kg AA-treated SCI groups (the AA-5, AA-100, and AA-200 group, respectively) at 1 week after injury. (**C**,**D**) Sizes of the lesion cavities (mm^2^) from the H&E staining results (**C**) and the total numbers of ED1-positive cells within sagittal sections from immunohistochemical images (**D**) for the control, AA-5, AA-100, and AA-200 groups (*n* = 4 per group); * *p* < 0.05 between groups by one-way analysis of variance (ANOVA) and Games–Howell post hoc tests. (**E**,**F**) BBB (**E**) and ladder (**F**) scores of the control, AA-5, AA-100, and AA-200 groups from 2 days (2 d) to 12 weeks (12 w) after injury (*n* = 7 per group); * *p* < 0.05 compared with the control groups by one-way ANOVA and Games-Howell post hoc tests. (**G**) Representative images of immunohistochemical staining of 5HT-positive axons (green) and GFAP-positive astrocytes (red) in the contused spinal cords of rats in the control, AA-5, AA-100, and AA-200 groups 12 w after injury. The left images are low-magnification images, while the right images are high-magnification images of the rostral (R), central (CT), and caudal (C) parts of the lesion cavities of contused spinal cords. (**H**,**I**) 5HT pixel density (**H**) and 5HT intensity (**I**) for the control, AA-5, AA-100, and AA-200 groups 12 w after injury (*n* = 4 per group). Yellow scale bar = 1000 µm; white scale bar = 100 µm; *p < 0.05 between groups by one-way ANOVA and Games-Howell post hoc tests.

Three different concentrations of AA (5, 100, and 200 mg/kg/day) were administered intraperitoneally to SCI models, and the models were observed for 12 weeks after treatment. H&E staining at 1 week revealed that the area of the cavity was smaller in the 100 mg/kg AA-treated SCI group (AA-100 group) than in the AA-200 group, but the area in the AA-100 group was not smaller than that in the control group (Figure 3A). There were fewer ED1-positive macrophages or microglia in the AA-100 group than in the control and AA-200 groups (Figure 3C). With regard to locomotor function during the 12 weeks after SCI, the AA-100 group started to show a significantly higher BBB score than the control group at 5 weeks (11.67 ± 0.71 vs. 10.22 ± 0.83; AA-100 group vs. control group), and the difference persisted until 12 weeks (12.33 ± 1.0 vs. 10.33 ± 0.87, respectively) (Figure 3E). The ladder test revealed a greater restoration of hind limb function in the AA-100 group than in the control group at 2 weeks (77.45 ± 11.03% vs. 93.20 ± 5.28%; AA-100 group vs. control group), and at all time points from 5 weeks (47.54 ± 17.83% vs. 73.54 ± 16.9%49%, respectively) until sacrifice at 12 weeks (23.50 ± 12.79 vs. 66.20 ± 16.66, respectively). After sacrifice, we performed IHC using a 5HT antibody to detect axonal sprouting within the lesion cavities of the control and AA-treated SCI model rats (Figure 3G). When the cavity was divided into three parts (rostral, central, and caudal parts), the 5HT pixel densities at the central and caudal parts were greater in the AA-100 group than in the control and other AA groups (the AA-5 and AA-200 groups) (Figure 3H). In addition, the 5TH intensity was higher in the AA-100 group than in the control group, whereas the 5HT intensity in the other AA groups was not different from that in the control group (Figure 3I). The intensity of NF200-positive neurofilaments within a lesion cavity was also higher in AA-100 group than in the control and other AA groups (Figure S2A,B).

These findings revealed that 100 mg/kg AA was the most effective concentration for reducing the numbers of ED1-positive macrophages or microglia, facilitating locomotor function recovery, and enhancing 5HT-positive axonal sprouting within the lesion cavity. Therefore, the AA-100 group was compared with the control group in subsequent molecular analyses.

### 3.4. AA Enhances 5hmC Formation by Upregulating Tet1 and Tet2

**Figure 4:**
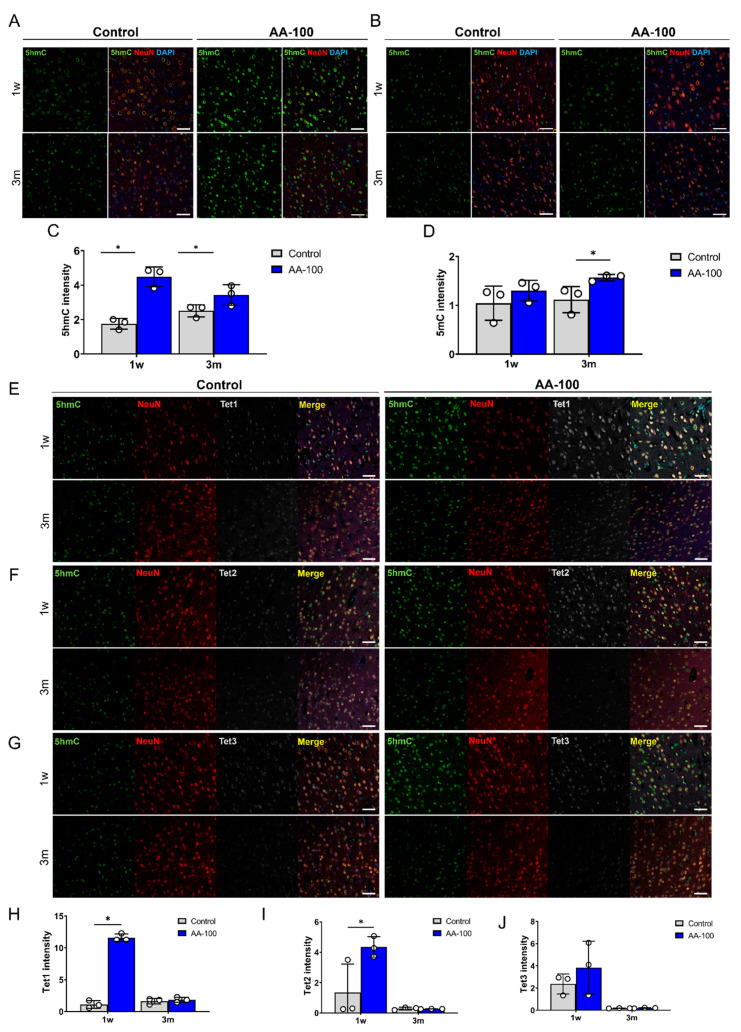
(**A**,**B**) Representative immunohistochemical images showing double staining of 5hmC (green) and NeuN (red) (**A**), and of 5mC (green) and NeuN (red) (**B**) in the primary motor cortexes of rats in the control and AA-100 groups 1 week (1 w) and 3 months (3 m) after injury. White scale bars = 50 µm. (**C**,**D**) The 5hmC (**C**) and 5mC (**D**) intensity in neurons in the primary motor cortexes of rats in the control (*n* = 3) and AA-100 (*n* = 3) groups 1 w and 3 m after injury; * *p* < 0.05 between the control and AA-100 groups at the same time point by Mann-Whitney U test. (**E**–**G**) Representative immunohistochemical images showing triple staining of Tet1 (gray), 5hmC (green), and NeuN (red) (**E**); Tet2 (gray), 5mC (green), and NeuN (red) (**F**); and Tet3 (gray), 5mC (green), and NeuN (red) (**G**) in the primary motor cortexes of rats in the control and AA-100 groups 1 w and 3 m after injury. White scale bars = 50 µm. (**H**–**J**) Tet1 (**H**), Tet2 (**I**), and Tet3 (**J**) intensity of 5hmC-stained neurons in the primary motor cortexes of rats in the control (*n* = 3) and AA-100 (*n* = 3) groups 1 w and 3 m after injury; * *p* < 0.05 between the control and AA-100 groups at the same time point by Mann-Whitney U test.

**Figure 5:**
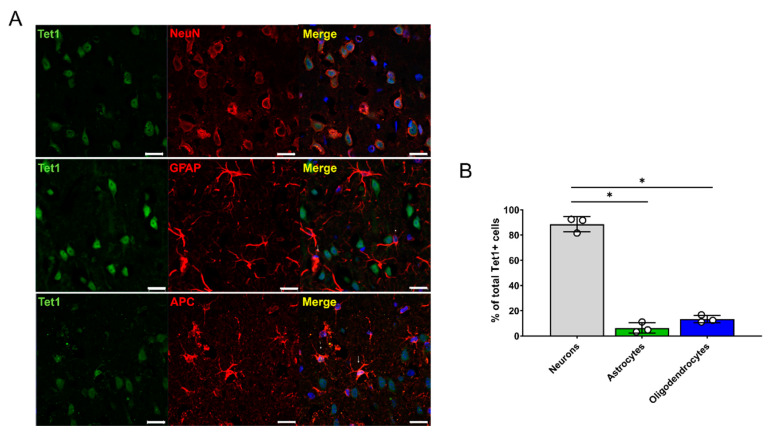
Distribution of Tet1 genes within neurons, astrocytes, and oligodendrocytes in the brain motor cortex 1 week after injury. (**A**) Representative immunohistochemical images of double staining of Tet1 (green) and NeuN (red), of Tet1 (green) and GFAP (astrocytes), and of Tet1 (green) and APC (red). White scale bars = 20 µm. (**B**) Percentages of NeuN-positive neurons, GFAP-positive astrocytes, and APC-positive oligodendrocytes among total Tet1-positive cells (*n* = 3 per cell type); * *p* < 0.05 between groups by one-way analysis of variance (ANOVA) and Games-Howell post hoc tests.

The histological and functional results obtained for the rats that received three concentrations of AA (5, 100, and 200 mg/kg) revealed that 100 mg/kg AA (in the AA-100 group) was superior to the other doses; therefore, histological and molecular changes within the brain motor cortex were evaluated for the AA-100 group. The 5hmC and 5mC levels in the brain motor cortex were determined using IHC in the control and AA-100 groups (Figure 4A–D). A relatively high intensity of 5hmC was detected in the AA-100 group at the 1- and 12-week time points (Figure 4A). The expression level of 5hmC was especially high in the AA-100 group at the 1-week time point. The average intensity of 5hmC was significantly higher in the AA-100 group than in the control group at 1 week and 3 months (Figure 4C). In contrast, the 5mC intensity levels were similar in both groups at 1 week but were higher in the AA-100 group than in the control group at 12 weeks (Figure 4B,D). Immunohistochemical staining of the TET family proteins (Tet1, Tet2, and Tet3) in the brain motor cortex was also performed at 1 week and 3 months following SCI (Figure 4E–J). We found that most Tet1-positive cells at 1 week after injury were NeuN-positive neurons (88.6 ± 2.9%), and relatively few cells were observed in adenomatous polyposis coli (APC)-positive oligodendrocytes (13.4 ± 6.1%) and GFAP-positive astrocytes (6.4 ± 4.2%) (Figure 5A,B). A similar pattern of Tet1 distribution to neurons, astrocytes, and oligodendrocytes pattern was shown at 12 weeks post-injury (Figure S3A,B). When we investigated colocalization of 5hmC-positive neurons with Tet1 using a triple staining method, we found that the Tet1 and Tet2 intensities within neurons were significantly higher in the AA-100 group than in the control group at 1 week but were not different between the two groups at 12 weeks (Figure 4E,H for Tet1 and Figure 4F,I for Tet2). The Tet3 intensity within neurons was not different between the two groups at 1 week and 3 months (Figure 4G,J).

### 3.5. AA Induces Changes in Gene Expression within the Brain after SCI

**Figure 6:**
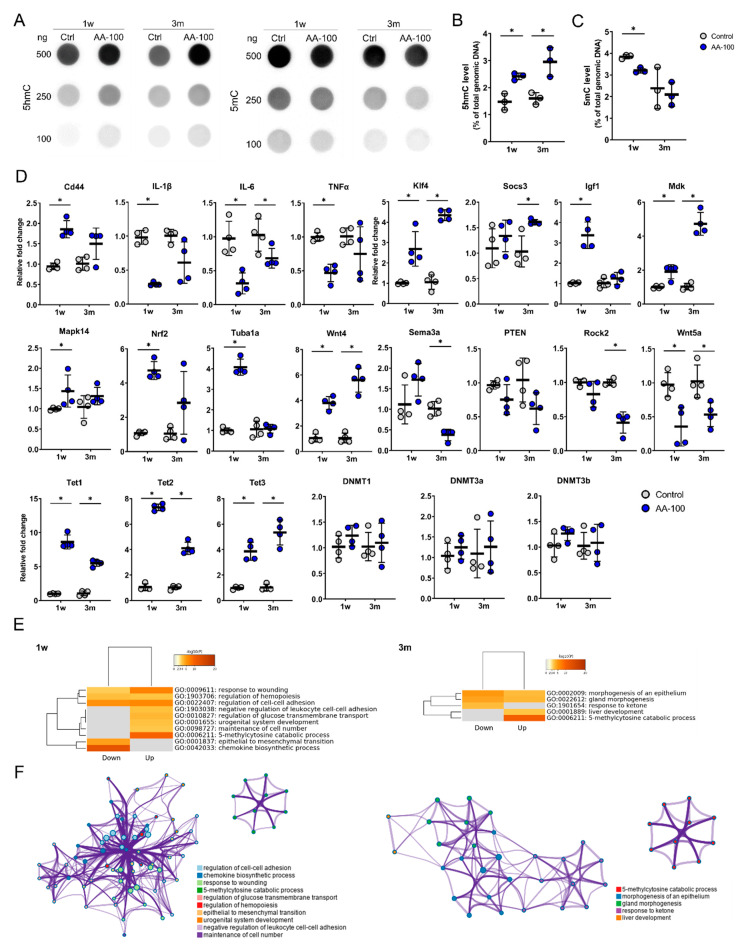
Dot blot and real-time PCR results for the primary motor cortexes of rats in the control and AA-100 groups at 1 week (1 w) and 3 months (3 m) after injury. (**A**) Representative results for 100, 250, and 500 ng of 5hmC and 5mC in the control and AA-100 groups 1 w and 3 m after injury. (**B**,**C**) Quantitative data from dot blot analysis of 250 ng of 5hmC (**B**) and 5mC (**C**) levels compared with the amount in total genomic DNA (%) in the control and AA-100 groups (*n* = 3 per group); * *p* < 0.05 between the control and AA-100 groups by Mann-Whitney U test at the same time point. (**D**) Quantitative data from real-time PCR for inflammation-related genes (Cd44, IL-1β, IL-6, and TNFα), regeneration-related genes (Klf4, Socs3, Igf1, Mdk, Mapk14, Nrf2, Tuba1a, and Wnt4), degeneration-related genes (Sema3a, PTEN, Rock2, and Wnt5a), TET family genes (Tet1, Tet2, and Tet3), and DNA methyltransferase genes (DNMT1, DNMT3a, and DNMT3b) at 1 w and 3 m after injury; * *p* < 0.05 between the control and AA-100 groups by Mann-Whitney U test at the same time point (*n* = 4 per group). (**E**) Gene Ontology (GO) functional enrichment analysis was performed for the differentially expressed genes between the control group and the AA-100 group at 1 w and 3 m after injury using Metascape. The values are colored by the negative log10-transformed *p*-values. Down = downregulated genes; Up = upregulated genes. (**F**) Network of the enriched GO terms for the upregulated genes at 1 w and 3 m after injury. The nodes are colored by cluster ID and the network was created using Metascape.

The levels of 5hmC and 5mC in total genomic DNA within the brain motor cortex were evaluated in the control and AA-100 groups using a DNA dot blot assay (Figure 6A). The 5hmC level was significantly higher in the AA-100 group than in the control group at 1 week and 3 months (Figure 6B), similar to the immunohistochemical results shown in Figure 3A,C; however, the 5mC level was lower in the AA-100 group than in the control group at 1 week (Figure 6C).

The qRT-PCR analysis of the brain motor cortex was performed to detect changes in gene expression 1 week and 3 months after 100 mg/kg AA treatment in SCI models (Figure 6D). The expression levels of inflammation-related genes, including IL-1β, IL-6, and TNFα, were significantly decreased at 1 week in the AA-100 group compared to the control group. In addition, the IL-6 gene was also downregulated at 3 months in the AA-100 group. Among the regeneration-related genes, Klf4, Igf1, Mdk, Mapk14, Nrf2, Tuba1a, and Wnt4 were upregulated at 1 week in the AA-100 group, and Klf4, Socs3, Mdk, and Wnt4 were upregulated at 3 months in this group. Wnt5a, a degeneration-related gene, was downregulated at 1 week and 3 months in the AA-100 group, while Sema3a and Rock2 were downregulated at 3 months. The mRNA levels of Tet1, Tet2, and Tet3 were significantly higher at both 1 week and 3 months in the AA-100 group than in the control group. However, DNMT1, DNMT3a, and DNMT3b gene expression was not changed at 1 week and 3 months after the application of 100 mg/kg AA. Functional enrichment analysis showed that the significantly upregulated Tet family genes at both 1 week and 3 months after injury in the AA-100 group were enriched for chemical reactions and pathways resulting in the breakdown of 5mC (GO: 0006211) (Figure 6E). The downregulated genes at 1 week in the AA-100 group (IL-1β, IL-6, TNFα, and Wnt5a) were enriched for the chemokine biosynthesis process (GO: 0045080) (Figure 6E). The upregulated and downregulated genes at 1 week that were enriched for the response to wounding (GO: 0009611) and regulation of cell-cell adhesion (GO: 0022407) terms were related and formed a network, while the 5mC catabolic process (GO: 0006211) was controlled independently 1 week and 3 months after injury (Figure 6F).

## 4. Discussion

One of the challenges in the treatment of SCI is the healing of devastating lesions through mainly intrinsic mechanisms. So far, some promising therapeutic approaches have been proposed by our group and others. One of our previous studies demonstrated that reduction in the ROS level by cerium oxide nanomaterials promote locomotor functions by altering gene expression [11]. In addition, another study revealed that transplantation of induced neural stem cells (iNSCs) into SCI models elicits beneficial effects, including amelioration of the inflammatory response and apoptosis in the injured area [10]. Both studies focused on promoting recovery from injury through local therapeutic administration. Given that 5hmC is increased around injured areas (Sun et al. 2018), in the present study, we sought to determine the changes in both 5mC and 5hmC in the brains of rats with SCI. Our analysis revealed that the 5hmC level in the motor cortex area was enhanced after SCI, as confirmed by both immunofluorescence and dot blot analysis. Intriguingly, the increase was prominently detected at 1 day to 1 week after SCI. Accordingly, Tet genes were found to be upregulated; *Tet1* and *Tet2* were upregulated in acute phases, whereas Tet3 was increased in chronic phases.

The *TET1* gene was initially discovered as a gene linked to acute myeloid leukemia (AML) that forms a fusion protein the histone H3 Lys 4 (H3K4) methyltransferase mixed-lineage leukemia (MLL) [19,20]. At that time, however, the function of the protein was enigmatic. In 2009, Tahiliani et al. discovered that TET1 catalyzes hydroxylation of 5mC to produce 5hmC [21], and Ito et al. showed that Tet proteins can further convert to 5hmC to 5fC and 5caC [22]. Since then, studies have revealed that Tet functions are involved in development processes, including cell reprogramming and differentiation, and in diseases, including amyotrophic lateral sclerosis and leukemia [23,24,25,26].

The levels and status of 5mC or 5hmC have been implicated in neurogenesis in the developing brain. Early studies showed that fine-tuned regulation of methylation status at the *Gfap* promoter and of *Gfap* expression by DNMTs impacts neuronal development [27,28]. In addition to 5hmC levels, 5mC levels were found to be increased at 1 day after SCI. However, the levels were reduced at 1 week, suggesting that each epigenetic mark plays a unique role in functional recovery after SCI. It is possible that the different dynamics between 5mC and 5hmC are caused by the different turnover rates of 5mC and its derivatives. It has been shown that the levels of 5hmC in the brain are ~10 times higher than those in other tissues, while much lower levels of 5caC and 5fC are detected in the brain [29,30].

It is also plausible that each Tet protein has a unique expression pattern and a different function depending on the cell type in the brain. Tet1-3 are highly expressed in the brain (Tet3 > Tet2 > Tet1), and they have been implicated in neuronal functions, as they colocalize with NeuN-positive neurons [31,32,33]. Similarly, we found that most Tet1-positive cells were NeuN-positive neurons (>88%) within the motor cortexes of rats (Figure 5). Tet1 knockout (KO) in mice enhances long-term depression and impairs memory extinction, whereas Tet2 KO increases neural stem cell proliferation and reduces differentiation potential [34,35]. Tet3 silencing in mouse neurons significantly elevates miniature glutamatergic excitatory postsynaptic current (mEPSC) amplitudes, whereas Tet3 overexpression decreases mEPSC amplitudes [36]. Furthermore, Tet3 has been shown to participate in neural progenitor cell (NPC) proliferation by regulating cyclin D1 expression [37]. The regulation of neural stem cell proliferation and differentiation by Tet2 is mediated by interaction with Foxoa3 and regulation of gene expression [35].

Although a study by Sun et al. showed that SCI induces elevations in 5hmC levels and Tet2 expression [38], that study did not reveal any genetic changes at the supraspinal level. In our study, 5hmC and 5mC levels were increased during the acute to subacute stages following SCI, and Tet1, Tet2, and Tet3 expression was also upregulated (Figure 1 and Figure 2). Furthermore, epigenetic changes that occur in the brain during recovery from SCI have been poorly understood thus far. Previous studies have revealed that peripheral nerve damage induces Tet protein activity for DNA demethylation and activates RAG expression to enhance the regeneration process [39,40,41]. However, it has been suggested that the epigenetic response to CNS damage might be different [39].

Damage to corticospinal tracts following SCI affects cortical gene expression at the acute stage, especially the expression of genes related to wounding, apoptosis, and neurogenesis [42]. In the present study, our analysis revealed that gene activation within the brain motor cortex reflects regeneration processes in the corticospinal tracts; proinflammatory cytokines (IL-1β, IL-6, and TNFα; Figure 2E) were upregulated at the very acute stage (1 h), and genes related to the production of ROS (TNFα, Klf4, Mapk14, and Sema3a; Figure 2E) were enhanced at the acute stage (1 day). This gene expression profile is highly reminiscent of one found previously in spinal cord tissue [43]. Fine-tuning the level of pro-inflammatory gene IL-1β produced from macrophage is critical to the proper process of axonal regeneration, as early upregulation of IL-1β promotes axon regeneration, whereas sustained high IL-1β level plays a detrimental role in the axon regeneration [44]. Our study also revealed that some apoptosis- and regeneration-related genes (Socs3, Mdk, and Mapk14; Figure 2E) in the brain cortex were downregulated at the chronic stage of SCI. It has been suggested that Socs3 might induce Bcl-2, which then inhibits apoptosis after SCI [45]. In addition, Mdk promotes neurite outgrowth and neuron survival in cell culture [46]; and Mapk14 critically regulates the immunological response and the production of specific cytokines and chemokines in astrocytes [47]. It is noteworthy that some of the regeneration-related genes also function as positive or negative regulators in inflammatory responses. For example, Socs3 plays an inhibitory role in pro-inflammatory cytokine-induced immune responses [48,49] In CNS, Socs3 is expressed in not only immune cells but other cell types, including neurons, astrocytes, oligodendrocytes, and microglia, and its expression is regulated by inflammatory cytokines [48]. Therefore, it is possible that the acceleration of axonal regeneration by the switched pattern of Socs3 expression results from combinatorial effects of regulatory mechanism in immune cells, glial cells, and neurons. Similarly to Pten function, Socs3 function is also essential for maintaining the proper number of astrocytes [50,51].

Given that SCI alters 5mC and 5hmC levels in the brain, we looked for potent supplements or drugs to further enhance DNA demethylation in order to promote axonal regeneration. Previous studies have reported that histological and functional recovery from SCI in models is promoted through administration of AA alone or in combination with other drugs [7,52,53]. However, one study has suggested that AA affects SCI only through limited mechanisms, such as through antioxidant and anti-inflammatory mechanisms that eliminate free radicals and ROS [53].

AA has several beneficial effects on the damaged CNS [7,54,55]. Oxidative stress is an important factor for pathogenesis of the injured brain and spinal cord [56,57], and excessive inflammation by stimulating the expression of proinflammatory cytokines and chemokines may exacerbate secondary injury after SCI [58]. Previous studies revealed that AA application following CNS injury prevented further neuronal damage and enhanced functional improvement [7,54], but other mechanisms such as epigenetic modification are still unknown.

We found that administration of the optimal concentration of AA (100 mg/kg) after SCI improved all the indexes of histological and functional recovery after SCI. We used three doses of AA in this study, where 5 mg/kg is the range of doses available to humans and the other two doses (100 and 200 mg/kg) are very high for clinical use (6 and 12 g for adult human). One previous study had reported 200 mg/kg of AA was more effective than 100 mg/kg of AA for histological and functional improvements, but their experiment was performed over only a 4-week period [7]. In addition, their SCI model using New York University (NYU) impactor was milder than our model, because BBB scores from the previous study and our study at 4 weeks were different (11.2 vs. 10.4, respectively). Our SCI models did not show any functional improvement during the 4-week period in either AA-100 or AA-200 groups, although some histological and epigenetic changes appeared in the AA-100 group at 1 week (Figure 3 and Figure 4). Although it is not clear why the AA-200 group did not show any effectiveness for the recovery of SCI, there is a possibility that complications may have occurred. Previous studies reported iron overload [59,60], which may lead to tumor formation, imbalance of the immune system, or increased risk of infection [61,62,63] and kidney stones [59,64], although we did not perform autopsies on animal models receiving AA.

In this study, we also found the expression of 5hmC, Tets, and regeneration-associated genes (RAGs) was enhanced with administration of 100 mg/kg of AA. Accumulating evidence suggests that AA is involved in the reaction of Fe^2+^- with α-ketoglutarate-dependent dioxygenases (Fe^2+^/α-KGDDs) as an electron donor. This reaction includes TET hydroxylases [65,66], Jumonji C (JmjC) domain containing histone demethylases (JHDMs) [67], DNA and RNA demethylases of the AlkB homolog (ALKBH) family [68], and prolyl hydroxylases [69]. DNA demethylation is also closely related to neuronal and synaptic plasticity [70], and AA can serve as a cofactor for TET dioxygenases, which catalyze the oxidation of 5mC to 5hmC and promote DNA demethylation [5,66]. In our study, DNMT levels did not change after AA application, whereas all Tet family members were upregulated at 1 week and 3 months after AA application (Figure 6D). Furthermore, 5mC levels either did not change or were decreased, whereas 5hmC levels were increased (Figure 6A–C). Changes in DNMTs following CNS lesions and AA application have not been identified previously; however, based on our results, AA does not affect global DNA methylation. Instead, 5mC changes due to AA might possibly occur in a selected set of genes.

Although AA-induced DNA demethylation may promote neuronal plasticity and regeneration, the relationships between DNA demethylation and activation of some RAGs induced by AA application are not clear in this study. SCI itself might not induce expression of RAGs due to impairment of local mRNA translation and a nonpermissive epigenome [39]. The antioxidant effects of AA might also attenuate the inflammatory response and concomitantly reduce lesion cavity size (Figure 3A–D); however, epigenetic changes that promote axonal sprouting adjacent to the lesion cavity (Figure 3G–I) might also contribute to functional recovery (Figure 3E,F). Nevertheless, our study cannot exclude that the beneficial effect of AA on SCI recovery at the acute phase might be due to the protective role of AA alone from oxidative damage around the wounded area. Another limitation of this study was that the number of samples used for some analyses was small, while variations between samples were large, making it difficult to reach a clearer conclusion, which should be investigated through further studies.

In this study, we sought to detect epigenetic changes following SCI at 5 different time points from the very acute to chronic stages and used AA to enhance DNA demethylation for the first time. However, only overall epigenetic changes in the brain motor cortex were identified; therefore, the epigenetic changes in the identified target genes after SCI or treatment should be confirmed in further studies to elucidate the mechanisms by which epigenetic modifications contribute to spinal cord regeneration or plasticity. Given that non-neuronal cells also express Tet proteins, follow-up studies are necessary to determine biological significances of Tet protein expression and change of 5hmC, if any, in the cell types during SCI recovery.

In conclusion, we found that epigenetic status in the brain motor cortex was changed after SCI. In addition, the optimal concentration of AA induced epigenetic modification and may contribute to functional recovery after SCI.

## Figures and Tables

**Table 1:** Primer sequences used for real-time PCR gene expression analysis.

Gene	Forward Primer	Reverse Primer
*Cd44*	gctatctgtgcagccaacaa	aagaggagctgaggcattga
*IL-1β*	cacctctcaagcagagcacag	gggttccatggtgaagtcaac
*IL-6*	accacccacaacagaccagt	cagaattgccattgcacaac
*TNF-α*	ctcaagccctggtatgagcc	ggctgggtagagaacggatg
*Mapk14*	gagctgttgaccggaagaac	tgagataagcagggggtgtc
*Socs3*	ccccgctttgactgtgtact	ctgctcctgaacctcaaagg
*Igf1*	cacactgacatgcccaagac	gggaggctcctcctacattc
*Mdk*	cctgaagaaggctcggtaca	ctttcttggctttggccttt
*Tuba1a*	cactacaccattggcaagga	gctgtggaaaaccaagaagc
*Klf4*	ctttcctgccagaccagatg	ggtttctcgcctgtgtgagt
*Ntrk2*	caagctgacgagtttgtcca	gagccacatgatgtcacagg
*Sema3a*	ttctggatgaggaacggagt	tggccacacaatcttttgaa
*PTEN*	acaagaggccctggattttt	gggtcctgagttggaggagt
*Rock2*	agccccctgcaaagtttatt	caccaaccgactaacccatt
*Wnt4*	cctttgcagtgacaagagca	ctgaccactggaaaccctgt
*Wnt5a*	gcagcacagtggacaacact	tcatggcatttaccactcca
*DNMT1*	gtgtgcgggaatgtgctcgct	cagtggtggtggcacagcgt
*DNMT3a*	agcaaagtgaggaccattaccacca	tgtgtagtggacagggaagcca
*DNMT3b*	tggcaaggatgacgttctgtggt	ctggcacactccaggaccttcc
*Tet1*	ggcttgcagacactgatgaa	gaaacacagtcgcctcttcc
*Tet2*	ggggttggagcaagtacaaa	cgggtgtgtgtcatttgaag
*Tet3*	agtgggtgatccgaagacac	gccaggatcaagatgacgat
*GAPDH*	cactgagcatctccctcaca	gagggtgcagcgaactttat

## Supplementary Materials

The following are available online at https://www.mdpi.com/2073-4409/9/5/1310/s1, Figure S1: Quantitative data from dot blot analysis of 500 and 100 ng of DNA for 5hmC and 5mC, Figure S2: DAB staining analysis of NF200 in the injured spinal cord tissue 12 w after injury, Figure S3: Distribution of Tet1 genes within neurons, astrocytes, and oligodendrocytes in the brain motor cortex 12 week after injury.
