# Supplementary material for: Ascorbic Acid Promotes Functional Restoration after Spinal Cord Injury Partly by Epigenetic Modulation
Source: Cells. 2020 May 25;9(5):1310. doi: 10.3390/cells9051310 (PMC7290865; doi:10.3390/cells9051310)
Supplement: Supplementary file 1 [file cells-09-01310-s001.zip › 200524 Supplemental Materials_Hyun.docx]

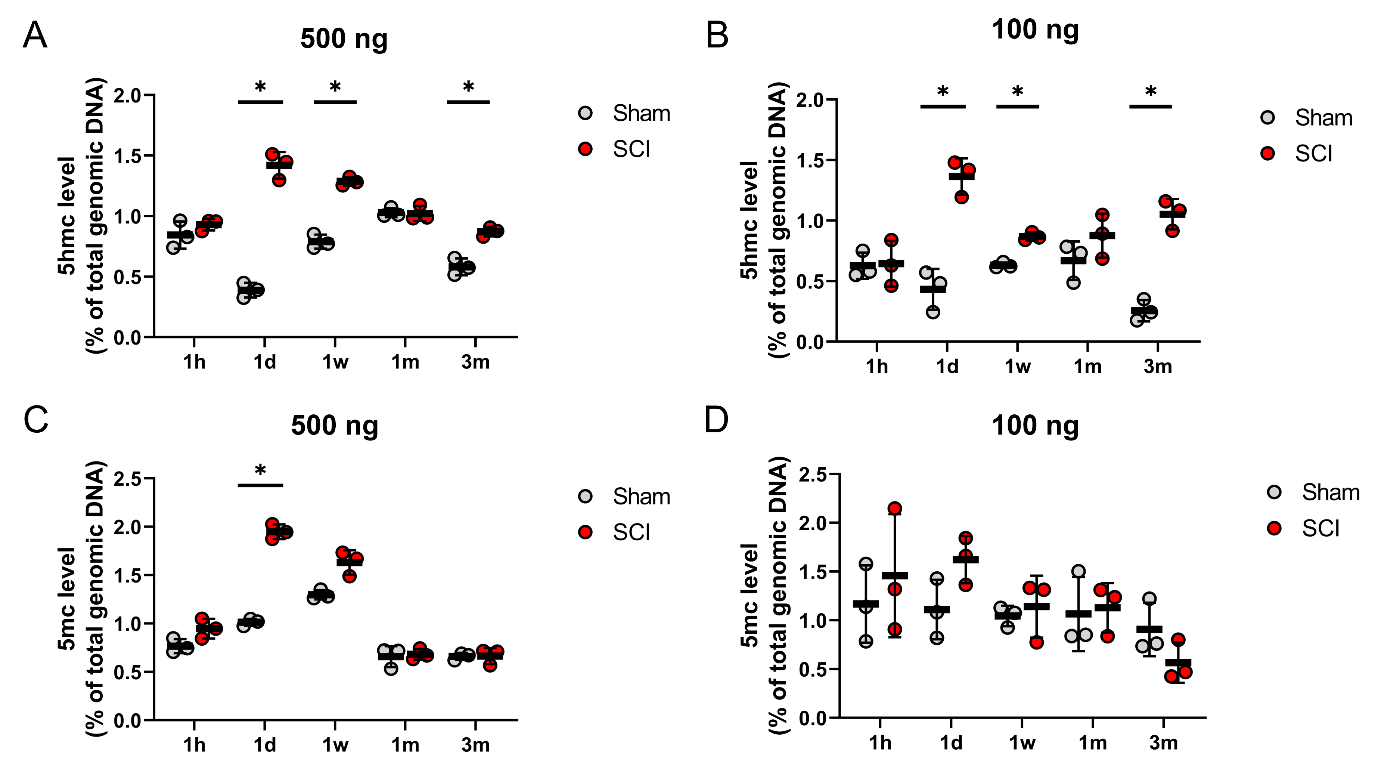


**Supplemental Figure 1. A-D.** Quantitative data from dot blot analysis of 500 ng (**A**) and 100 ng (**B**) of DNA for 5hmC, and 500 ng (**C**) and 100 ng (**D**) of DNA for 5mC compared with the amount in total genomic DNA (%) in the sham and SCI groups (n=3 per group). *p<0.05 between the sham control and SCI groups by Mann-Whitney U test at the same time point.


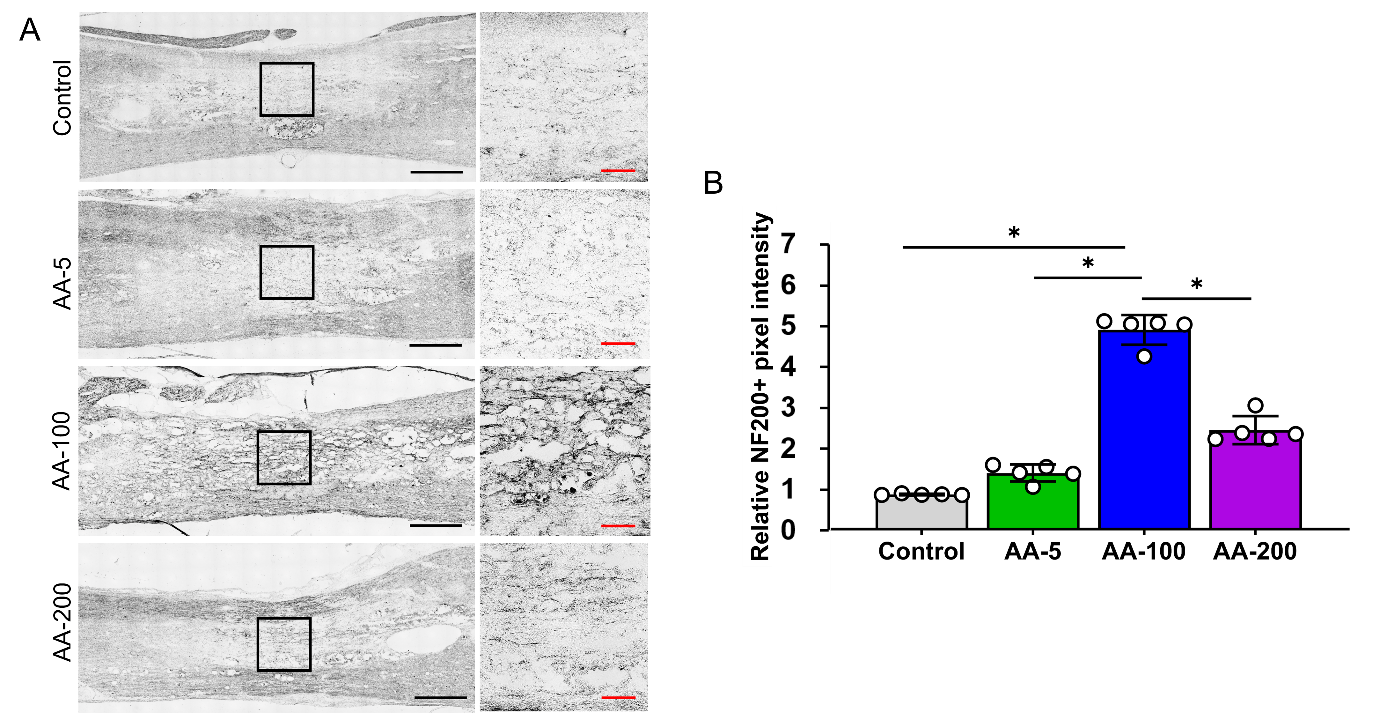


**Supplemental Figure 2.** DAB staining analysis of NF200 in the injured spinal cord tissue 12 w after injury. **A.** Representative images of NF200-positive axons in the control, AA-5, AA-100, and AA-200 groups. **B.** The relative intensity of NF200-positive axons of the control, AA-5, AA-100, and AA-200 groups. Black scale bars = 1 mm, red scale bar = 200 μm.


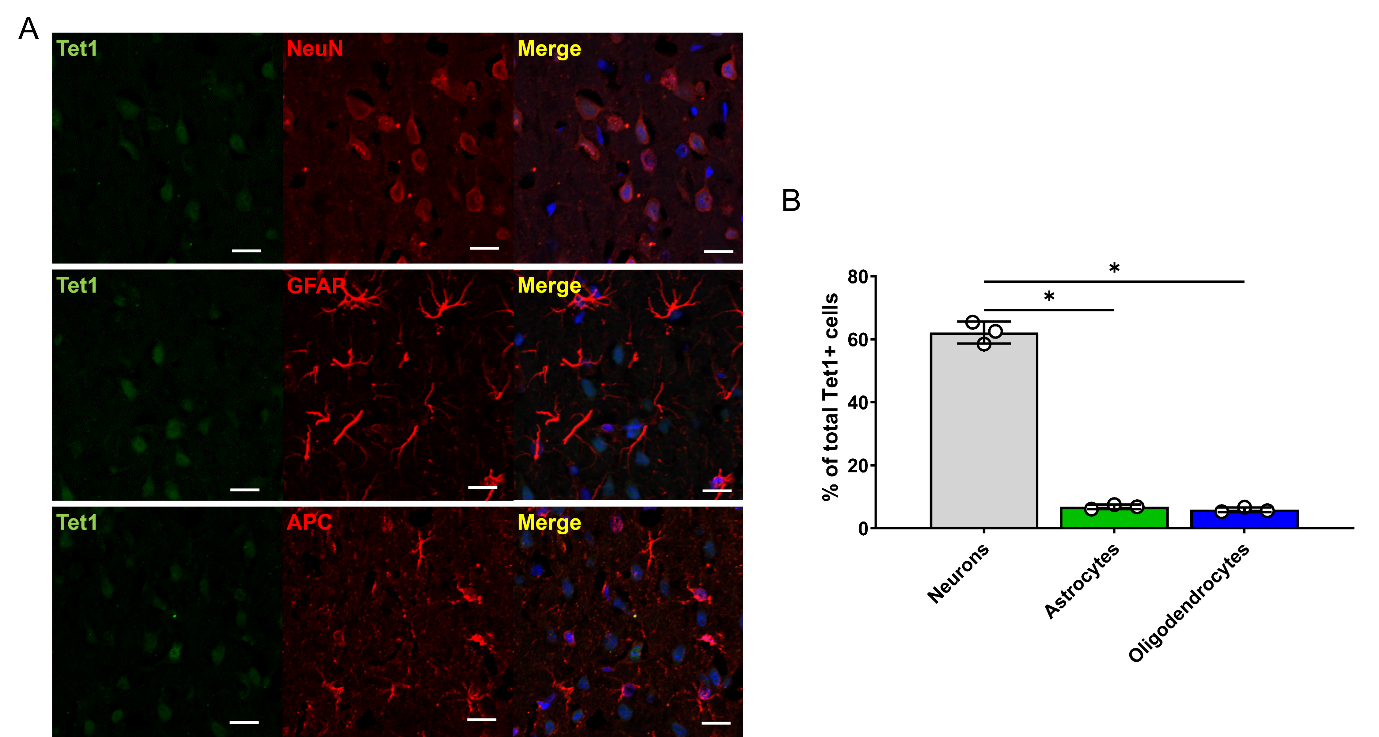


**Supplemental Figure 3.** Distribution of Tet1 genes within neurons, astrocytes, and oligodendrocytes in the brain motor cortex 12 week after injury. **A.** Representative immunohistochemical images of double staining of Tet1 (green) and NeuN (red), of Tet1 (green) and GFAP (astrocytes), and of Tet1 (green) and APC (red). White scale bars = 20 µm. **B.** Percentages of NeuN-positive neurons, GFAP-positive astrocytes, and APC-positive oligodendrocytes among total Tet1-positive cells (n=3 per cell type). *p<0.05 between groups by one-way analysis of variance (ANOVA) and Games-Howell post hoc tests.
