# Supplementary figures and images for: Ascorbic Acid Promotes Functional Restoration after Spinal Cord Injury Partly by Epigenetic Modulation
Source: Cells. 2020 May 25;9(5):1310. doi: 10.3390/cells9051310 (PMC7290865; doi:10.3390/cells9051310)

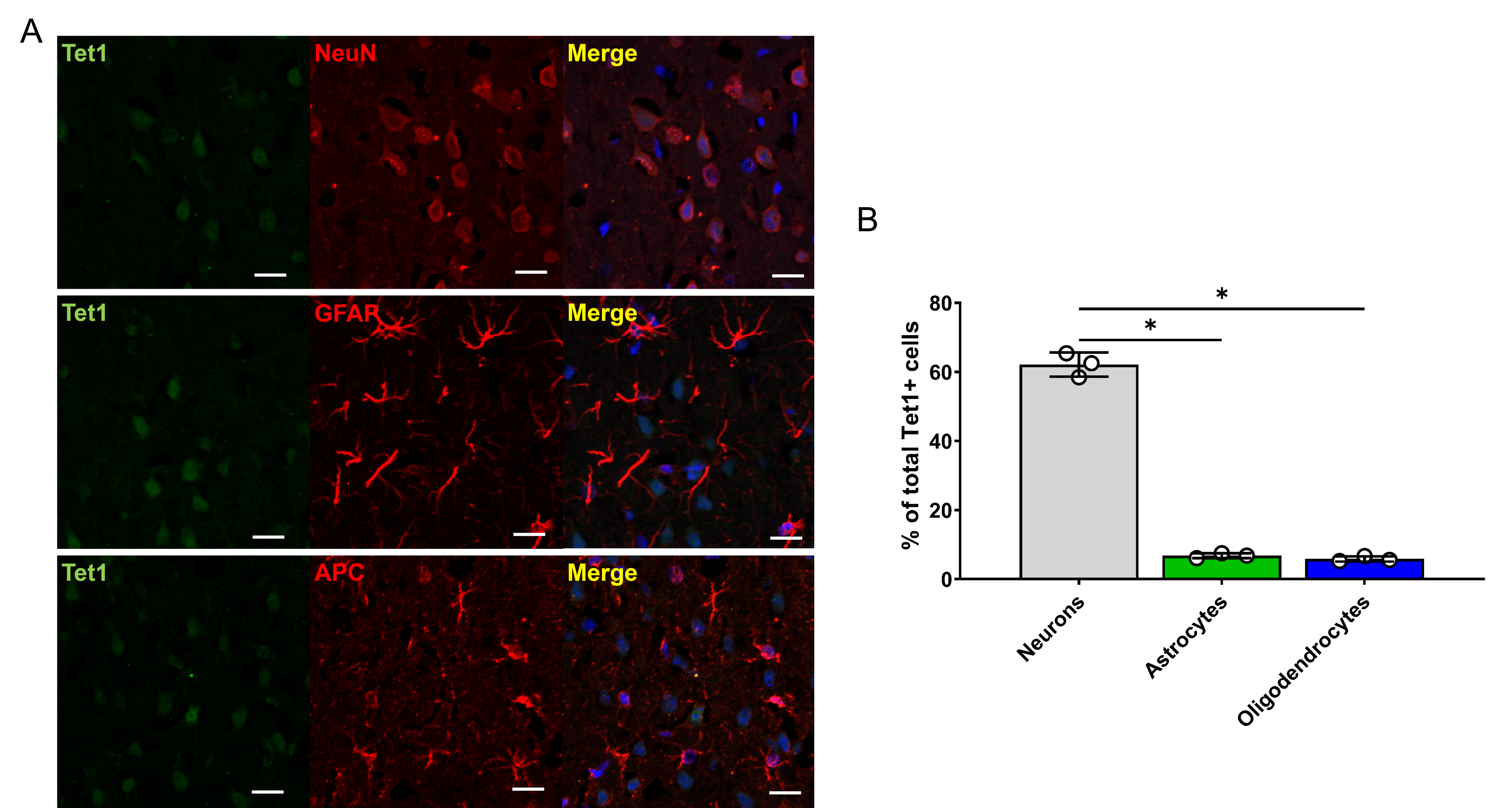

Supplement: Supplementary file 1 [file cells-09-01310-s001.zip › Supplemental Figure 3.png]

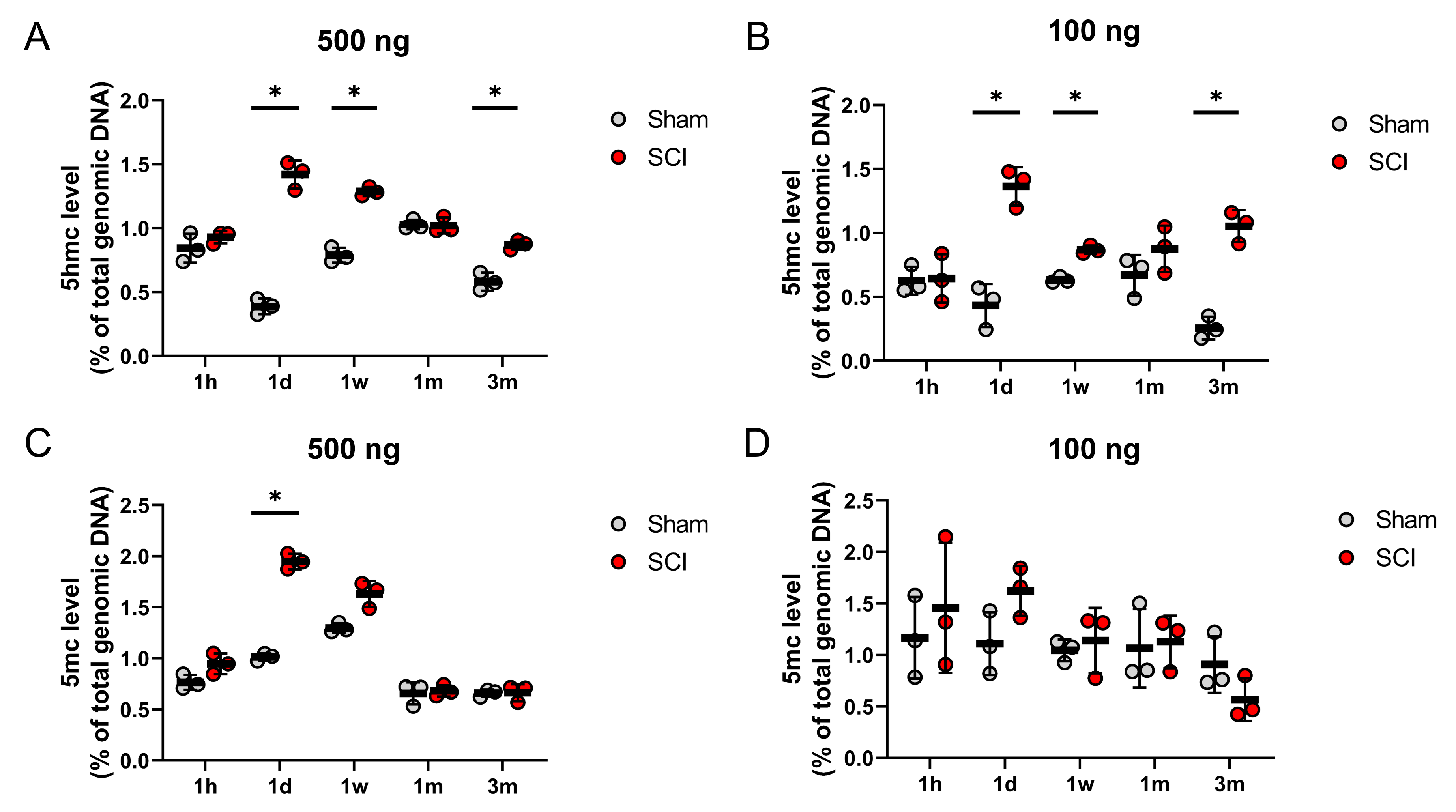

Supplement: Supplementary file 1 [file cells-09-01310-s001.zip › Supplemental Figure 1.png]

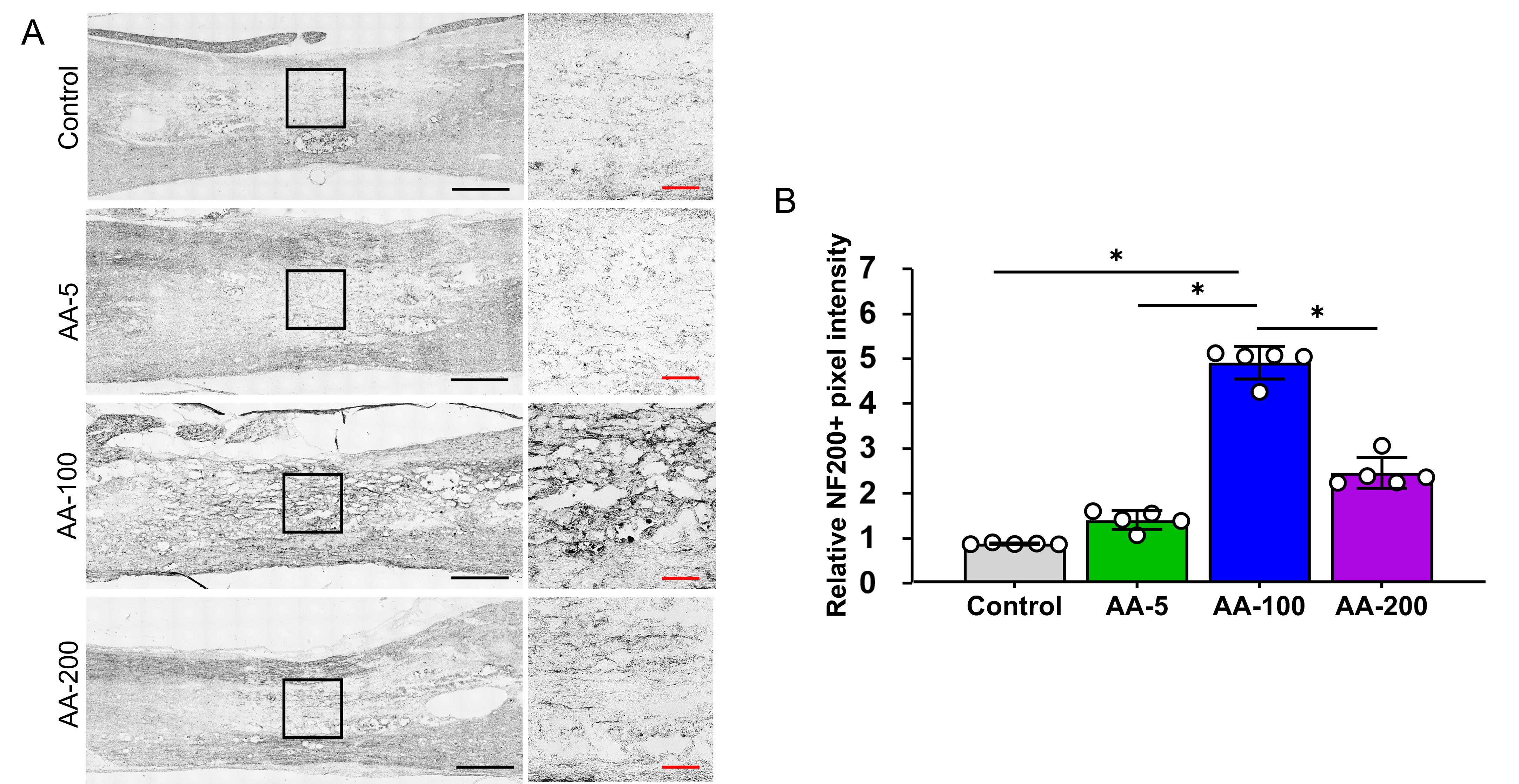

Supplement: Supplementary file 1 [file cells-09-01310-s001.zip › Supplemental Figure 2.png]
